# Tele-Monitoring of Cancer Patients’ Rhythms during Daily Life Identifies Actionable Determinants of Circadian and Sleep Disruption

**DOI:** 10.3390/cancers12071938

**Published:** 2020-07-17

**Authors:** Francis Lévi, Sandra Komarzynski, Qi Huang, Teresa Young, Yeng Ang, Claire Fuller, Matei Bolborea, Julia Brettschneider, Joanna Fursse, Bärbel Finkenstädt, David Pollard White, Pasquale Innominato

**Affiliations:** 1 Cancer Chronotherapy Team, Warwick Medical School, Coventry CV4 7AL, UK; S.Komarzynski@warwick.ac.uk (S.K.); Q.Huang.6@warwick.ac.uk (Q.H.); M.Bolborea@warwick.ac.uk (M.B.); Joanna.fursse@nhs.net (J.F.); Pasquale.Innominato@wales.nhs.uk (P.I.); 2 European Laboratory U935, Institut National de la Santé et de la Recherche Médicale (INSERM), Paris-Saclay University, 94801 Villejuif, France; B.F.Finkenstadt@warwick.ac.uk; 3 Hepato-Biliary Centre, Paul Brousse Hospital, Assistance Publique Hôpitaux de Paris (AP-HP), 94800 Villejuif, France; 4 Department of Statistics, University of Warwick, Coventry CV4 7AL, UK; Julia.Brettschneider@warwick.ac.uk; 5 Mount Vernon Cancer Centre, East and North Hertfordshire NHS Trust, Northwood, Middlesex HA6 2RN, UK; Teresa.young2@nhs.net; 6 Salford Royal NHS Foundation Trust, Salford M6 8HD, UK; Yeng.Ang@srft.nhs.uk; 7 Gastrointestinal Sciences, Division of Diabetes, Endocrinology and Gastroenterology, Faculty of Biology, Medicine and Health, University of Manchester, Manchester M13 9PL, UK; 8 North Wales Cancer Treatment Centre, Ysbyty Gwynedd, Betsi Cadwaladr University Health Board, Bangor LL57 2PW, UK; Claire.Fuller2@wales.nhs.uk; 9 Division of Sleep and Circadian Disorders, Brigham and Women’s Hospital, Harvard Medical School, Boston, MA 02130, USA; dpwhite@partners.org; 10 Philips Respironics, Murrysville, PA 15668, USA

**Keywords:** gastro-intestinal cancer, circadian rhythms, sleep, circadian regulation, biomarkers, patient-reported outcome measures, cortisol, melatonin, age, sex

## Abstract

The dichotomy index (I < O), a quantitative estimate of the circadian regulation of daytime activity and sleep, predicted overall cancer survival and emergency hospitalization, supporting its integration in a mHealth platform. Modifiable causes of I < O deterioration below 97.5%—(I < O)_low_—were sought in 25 gastrointestinal cancer patients and 33 age- and sex-stratified controls. Rest-activity and temperature were tele-monitored with a wireless chest sensor, while daily activities, meals, and sleep were self-reported for one week. Salivary cortisol rhythm and dim light melatonin onset (DLMO) were determined. Circadian parameters were estimated using Hidden Markov modelling, and spectral analysis. Actionable predictors of (I < O)_low_ were identified through correlation and regression analyses. Median compliance with protocol exceeded 95%. Circadian disruption—(I < O)_low_—was identified in 13 (52%) patients and four (12%) controls (*p* = 0.002). Cancer patients with (I < O)_low_ had lower median activity counts, worse fragmented sleep, and an abnormal or no circadian temperature rhythm compared to patients with I < O exceeding 97.5%—(I < O)_high_—(*p* < 0.012). Six (I < O)_low_ patients had newly-diagnosed sleep conditions. Altered circadian coordination of rest-activity and chest surface temperature, physical inactivity, and irregular sleep were identified as modifiable determinants of (I < O)_low_. Circadian rhythm and sleep tele-monitoring results support the design of specific interventions to improve outcomes within a patient-centered systems approach to health care.

## 1. Introduction

Chronic disease patients represent the largest burden for our health care system, and are at highest risk of acute complications, as currently seen in the COVID-19 pandemic [1,2]. There is a need for a comprehensive patient-centered health care system, which gathers and analyzes patients’ data during daily life, and provides them and their families and hospital physicians with information enabling shared decision-making procedures [3,4]. Such a “domomedicine” system will anticipate and prevent emergency hospitalizations through proactive interventions, based on early warning signals extracted from critical health functions dynamics tele-monitoring. This presents an immense opportunity for progress through circadian medicine to integrate our rapidly increased understanding of how molecular circadian clocks work and control cellular and organismic physiologies over the course of 24 h [5]. Dedicated and non-invasive biomarkers, such as rest-activity, body temperature, sleep, cortisol, and melatonin, can help to diagnose circadian alterations, and monitor disease or treatment effects on the circadian timing system (CTS), especially for cancer patients [6]. The CTS links a central circadian pacemaker, the hypothalamic suprachiasmatic nuclei, to the widespread cellular genetic clocks through the generation of an array of physiological and hormonal rhythms [7]. Circadian oscillations are generated in each mammalian cell by a molecular oscillator involving some 15 specific genes regulating each other through transcriptional and post transcriptional feedback loops [8,9]. The alternation of days and nights, social interactions, and meal timing, i.e., the daily routines, help to calibrate the period of the CTS, to enhance its coordination and robustness, and to reset its timing [6].

In cancer patients, strong links have been shown between overall survival and rest-activity, salivary cortisol, and temperature circadian biomarkers [10,11,12,13,14]. The disruption of the circadian rest-activity rhythm was shown to be a significant predictor for emergency hospitalization three days later in cancer patients on chemotherapy [15,16]. Overall survival, as well as global and specific domains of health-related quality-of-life in large cohorts of cancer patients were predicted by the dichotomy index (I < O), defined as the relative amount of activity In-bed that is below the median activity out-of-bed [11,12,13]. Previous research had further shown that I < O ranged from 50 to 100% in over 500 patients with advanced or metastatic cancer. The median I < O value has consistently been close to 97.5% in cancer patients, compared to 99.5% in healthy subjects (*p* < 0.0001), thus highlighting the clinical relevance of this parameter [17]. Thus, I < O values below 97.5%, have been associated with more prevalent and severe systemic symptoms [18], poorer global and specific domain measured health-related quality of life, and shorter progression-free and overall survival [10,12,13,19,20,21,22] For instance, in 436 patients with metastatic colorectal cancer, the median overall survival rate was 21.6 months (range: 17.8–25.5) for those with I < O above the median value of 97.5%, compared to 11.9 months (range: 10.4–13.3) for those with a lower I < O (Log-rank *p* < 0.001). Multivariate analyses retained continuous I < O as a joint predictor of both overall survival and progression-free survival (*p* < 0.001) [12]. The integration of such information on circadian rhythms into daily oncologic practice could indeed advance personalized and precision cancer medicine.

This can now be done, since the monitoring of multiple physiological rhythms, jointly with symptoms and other data, has become possible through mobile e-Health platforms linking Bluetooth Low Energy (BLE) connected sensors and other devices to a central server via a General Packet Radio Service (GPRS) gateway. Such a domomedicine platform proved to be acceptable and technically reliable in field studies involving 223 people, including shift workers and cancer patients [23]. Appropriate statistical methods were developed for estimating CTS function in an individual subject during their daily life [24,25]. Here, we identify the main actionable determinants of circadian and sleep disruption through tracking two circadian biomarkers in real time in two clinical studies involving a pooled sample of size 58 comprising both cancerous and non-cancerous individuals. Our findings support the crucial potential of a comprehensive patient-centered digital therapeutics system for boosting medical progress.

## 2. Results

### 2.1. Study Flow, Participants’ Characteristics, Compliance, and Data Quality

#### 2.1.1. Cancer Patients

**Figure 1:**
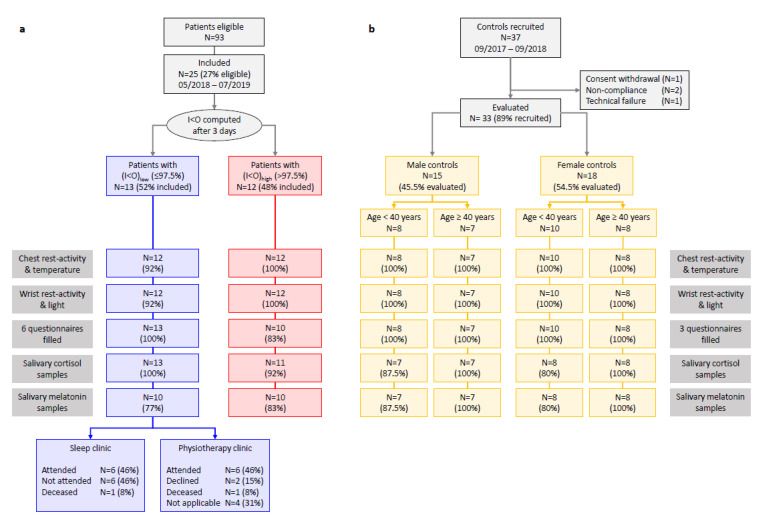
Consort diagram. Flow diagrams of both studies showing the enrollment of subjects and the variables that were measured. (**a**) Cancer patients, as categorized according to the a priori defined threshold of the dichotomy index I < O. Chest rest-activity and temperature not available for one patient due to logistics issues. Wrist rest-activity and light exposure pattern not available for one patient due to patient non-compliance. Chronotype not available for one patient. Cortisol, melatonin, diary and the M.D. Anderson Symptom Inventory (MDASI) questionnaires not provided by one patient. No or insufficient melatonin or cortisol samples for two patients. (**b**) Controls, stratified according to age and sex (parameters reported here are the ones which are common to both studies). Three controls did not provide any salivary samples as they were recruited before inclusion of salivary samples collection to the protocol.

Similar numbers of cancer patients were classified as either (I < O)_low_ (13 patients) or (I < O)_high_ (12 patients) based on the initial 72 h of accelerometry recording at chest level (Figure 1a). There were 21 males (84%) and four females (16%) with an overall median age of 66 years (range 40 to 82) and a good WHO performance status (Table 1). Most patients had primary colorectal cancer, metastatic disease, and prior chemotherapy. Seventeen of the 25 cancer patients (65%) had at least one additional comorbidity, including 58% in the (I < O)_high_ group and 77% of the patients in the (I < O)_low_ group. Twenty-two cancer patients (88%) were taking at least one concurrent medication, which were mostly related to the treatment of associated cardiovascular (*N* = 11, 44%), endocrine/metabolic (*N* = 10, 40%), and/or respiratory (*N* = 6, 24%) pathologies. The distribution of performance status, comorbidities and associated medications intakes did not differ significantly between both I < O groups. Three patients (12%) were suffering from anxiety and/or depression according to results from the hospital anxiety and depression scale (HADS) questionnaire. Overall subjective sleep quality, reported with the Pittsburgh sleep quality index (PSQI), was good or fairly good (with a global score ≤ 10) for 18 patients. No significant differences were found for patient characteristics as well as for PSQI and HADS scores according to I < O category.

#### 2.1.2. Controls

Of the 33 evaluated participants (89% of those included), there were 15 males and 18 females, including 15 participants aged 40 to 78 years of age (Figure 1b, Table 1). Three control subjects (33%) had past medical conditions and eight (24%) reported a mild and medically controlled pathologic condition (24.3%). Eight control subjects took one or more daily medications. No control subject took any medication known to influence circadian rhythms, such as beta-blockers, melatonin or agonists, or glucocorticoids. A single participant was taking low dose aspirin for thrombosis prevention.

The cancerous participants tended to have a larger BMI, as compared to the controls. On the other hand, the distribution of chronotypes assessed with the Morningness-Eveningness Questionnaire (MEQ) was strikingly similar for patients in both I < O categories as well as the control group, with morning types being predominant. On average, the control participants were younger than the cancer patients with (I < O)_high_ (*p* < 0.0001). The majority of the controls were employed or students while the patients in the (I < O)_high_ group were mostly retired or not working (*p* = 0.002). No ongoing medical condition was documented in 76% of the controls, while 58% of the patients in the (I < O)_high_ group had one or more medical conditions in addition to their cancer (*p* = 0.003).

#### 2.1.3. Compliance

The median overall compliance with the study protocol was 98% in cancer patients and 96% in controls. It exceeded 96% for 75% of the cancer patients irrespective of I < O category, and 93% for 75% of the controls irrespective of sex or age. These results highlighted the exceptional quality of the tele-transmitted and other datasets observed during daily life, for both advanced cancer patients outside the hospital setting and controls.

### 2.2. Tele-Transmitted Rest-Activity Patterns

**Figure 2:**
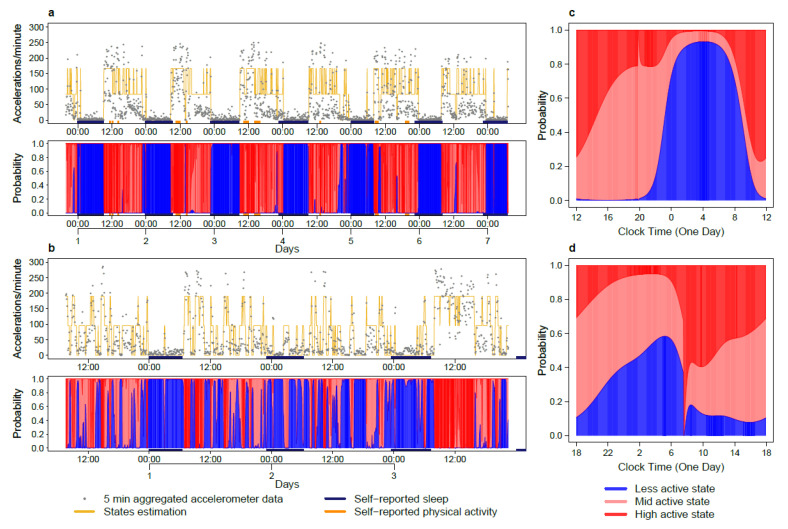
Rest-activity states estimation. (**a**) Rest-activity plots of one patient in the (I < O)_high_ group, with an I < O value of 99.1%. Top row: time series of rest-activity with yellow line indicating the most likely state using hidden Markov modelling (HMM) decoding. Bottom row: state probability plot showing the cumulative probabilities of low (blue), intermediate (pink) and high (red) active states. The color bars represent the patient’s self-reported sleeping and physical exercise spans. (**b**) Rest-activity plots of an example patient in the (I < O)_low_ group, with an I < O value of 86.5%. (**c**) Circadian state probability plot from harmonic HMM of the rest-activity pattern of the patient shown in (**a**). The panel shows the periodic time profile of the three state probabilities plotted as a cumulative manner analogous to the state probability plot with same color coding as in the bottom row of (**a**). (**d**) Circadian state probability plot from harmonic HMM of the rest-activity pattern of the patient shown in (**b**).

**Figure 3:**
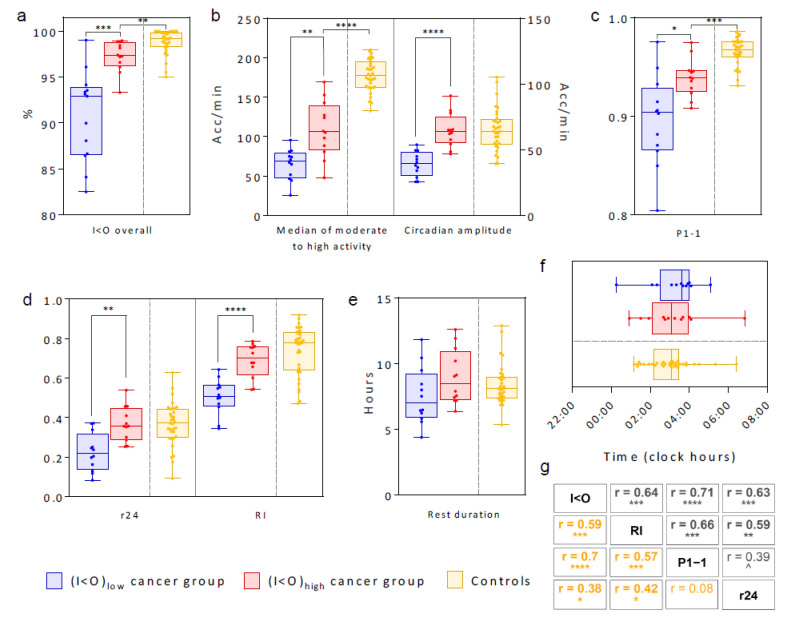
Box plots comparing the main parameters of the tele-transmitted rest-activity patterns in (I < O)_low_ and (I < O)_high_ cancer groups, and in the controls. (**a**) Box plots of I < O values computed over 7 days for each I < O patient group and controls. (**b**) Median of MA and HA activity states and circadian amplitude (spectral analysis). (**c**) P1-1 (HMM). (**d**) R24 and Rhythm Index. (**e**) Estimated rest duration. (**f**) Center-of-rest time. (**g**) Correlation matrices of the rest-activity rhythms parameters for cancer patients (black) and controls (yellow). In (**a**–**e**), the bars represent the extremes and bold lines inside the box plots plot median levels. Values for each individual are plotted as jittered dots to better show the distribution. Levels of significance: **** *p* ≤ 0.0001; *** 0.0001 < *p* ≤ 0.001; ** 0.001 < *p* ≤ 0.01; * 0.01< *p* ≤0.05; ^ 0.05 < *p* ≤0.1 (two-tailed Welch’s modified *t*-test (**a**–**e**); Pearson’s correlation (**f**)).

**Figure 4:**
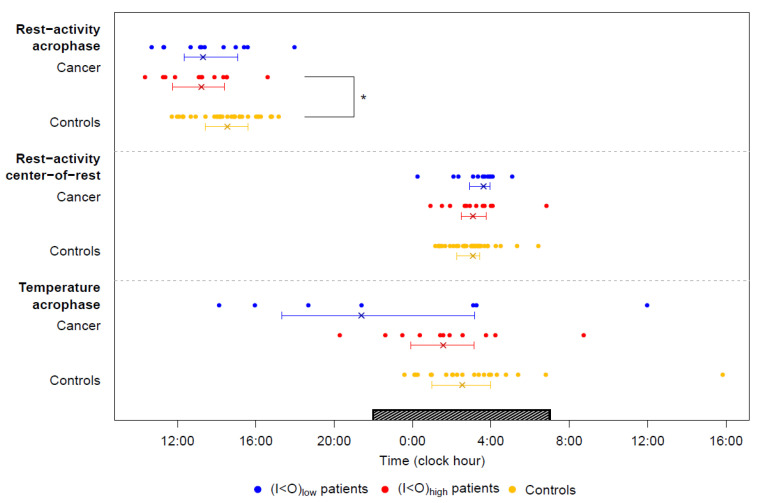
Daily timing of the tele-monitored circadian biomarkers. Median, interquartiles, and individual timings of each circadian biomarker in (I < O)_low_ and (I < O)_high_ patients and in controls. Median values are marked by a cross; horizontal bars represent interquartile range. Timings for each individual are plotted as dots (for rest-activity, 12 patients for each I < O group, and 33 controls; for temperature, 7 and 11 patients in the (I < O)_low_ and (I < O)_high_ groups, respectively, and 21 controls). The dark bar on the abscissa represents the night span (22:01 to 7:00). Chest surface temperature acrophase are shown for the participants displaying a significant circadian rhythm. Levels of significance: * 0.01< *p* ≤0.05 (two-tailed Welch’s modified *t*-test).

The circadian pattern in rest-activity was characterized by large inter-subject differences for both cancer patients and controls. For cancer patients, the (I < O)_low_ group clearly had worse chest rest-activity rhythm parameters than the (I < O)_high_ group (Figure 2). As could be expected, the median I < O values, computed over the full 7-day study span differed significantly between both groups (*p* < 0.0001, Figure 3a). In the (I < O)_low_ group, compared to the (I < O)_high_ group, there was a nearly 40% reduction in activity in moderately (MA) or highly active (HA) states (*p* = 0.0014), and circadian amplitude in activity (*p* < 0.0001), a significant 4% decrease in the estimated probability of staying in the rest state (P1-1) (*p* = 0.01), a circa 40% reduction in the estimated autocorrelation coefficient (r24) (*p* = 0.0012), and a circa 30% decrease in the rhythm index (RI) (*p* < 0.0001) (Figure 3b–d). On the other hand, rest durations (Figure 3e), center-of-rest times, and circadian acrophase of rest-activity were similar between both groups (Figure 4).

The I < Os of the cancer patients, including those in the (I < O)_high_ group, were significantly worse than those of the controls (median values, 99.2 vs. 97.3%, *p* = 0.008) (Figure 3a). This was also the case for the levels of the activity (177.3 vs. 106.1 acc/min in HA states, *p* < 0.0001), the rest probability P1-1 (0.97 vs. 0.94, *p* = 0.005), but not for circadian amplitude of activity, r24, RI, center-of-rest and the rest duration (Figure 3b–f). The four parameters (I < O, P1-1, RI, r24) were strongly correlated with each other in pairwise comparisons, except for r24 and P1-1 (Figure 3g). The latter is not surprising, as P1-1 has a focus on rest periods only, while r24 is also influenced by regular recurrence during the active periods.

### 2.3. Tele-Transmitted Chest Surface Temperature Patterns

**Figure 5:**
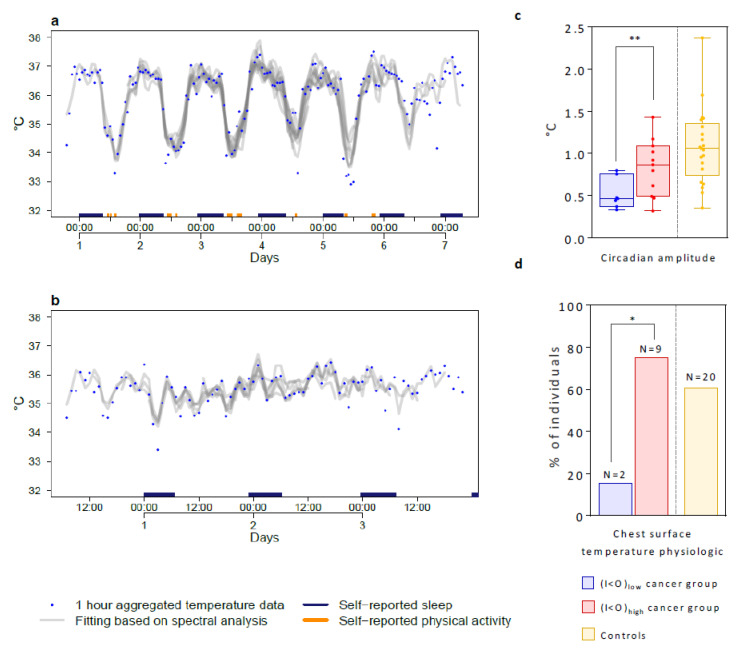
Chest surface temperature patterns and boxplots comparing the parameters of the tele-transmitted temperature patterns in (I < O)_low_ and (I < O)_high_ cancer groups, and in controls. (**a**) Time plot of chest surface temperature and curve fitting using spectral analysis for an example (I < O)_high_ patient with an I < O value of 99.1%. The color bars on the abscissa represent the patient’s self-reported sleeping and physical exercise spans. (**b**) Time plot of chest surface temperature for an example (I < O)_low_ patient with an I < O value of 86.5%. (**c**) Box plots showing the distribution of the chest surface temperature circadian amplitude for the two I < O patient and control groups. Bars represent the extremes and bold lines inside the box plots plot median levels. Values for each individual are plotted as jittered dots to better show the distribution. (**d**) Bar graphs of the proportion of individuals with a physiologic chest surface temperature in each I < O patient group and controls. Levels of significance: ** 0.001< *p* ≤0.01 (two-tailed Welch’s modified *t*-test in (**c**)); * 0.01< *p* ≤0.05 (Fisher’s exact test in (**d**)).

The (I < O)_low_ cancer patients had markedly deteriorated temperature rhythm parameters compared to the (I < O)_high_ ones (Figure 5a,b). A dominant circadian rhythm in chest surface temperature was found for 92% of the patients in the (I < O)_high_ group, but only for 58% in the (I < O)_low_ group (*p* = 0.15), who also displayed an approximately 40% reduction in temperature circadian amplitude (*p* = 0.021) (Figure 5c). Furthermore, nine out of 12 patients in the (I < O)_high_ group (75%) had a physiologic circadian rhythm in temperature, with an acrophase at night between 22:01 and 07:00. This was the case only for two out of 12 patients who provided recordings in the (I < O)_low_ group (16.6%) (*p* = 0.0123) (Figure 5d), while the other ten patients (83.5%) had either an abnormal temperature rhythm (*n* = 5) or no detectable rhythmic pattern at all (*n* = 5).

We therefore concluded that the disruption of the circadian rhythm in chest surface temperature was strongly associated with that of the rest-activity circadian rhythm, thus, supporting a major and selective alteration of the central circadian coordination in cancer patients with a low I < O. In contrast, temperature circadian acrophase (Figure 4) and amplitude, as well as the relative rate of subjects with a physiologic circadian temperature rhythm, were similar between the (I < O)_high_ cancer patients and controls (*p* = 0.23, *p* = 0.49, and *p* = 0.49, respectively) (Figure 5c,d).

### 2.4. Lifestyle, Cortisol and Dim Light Melatonin Patterns

No significant or relevant differences were found for the external time cues that synchronize circadian rhythms both between the two I < O cancer groups, and between cancer patients and controls (Appendix A, Figure S1).

**Figure 6:**
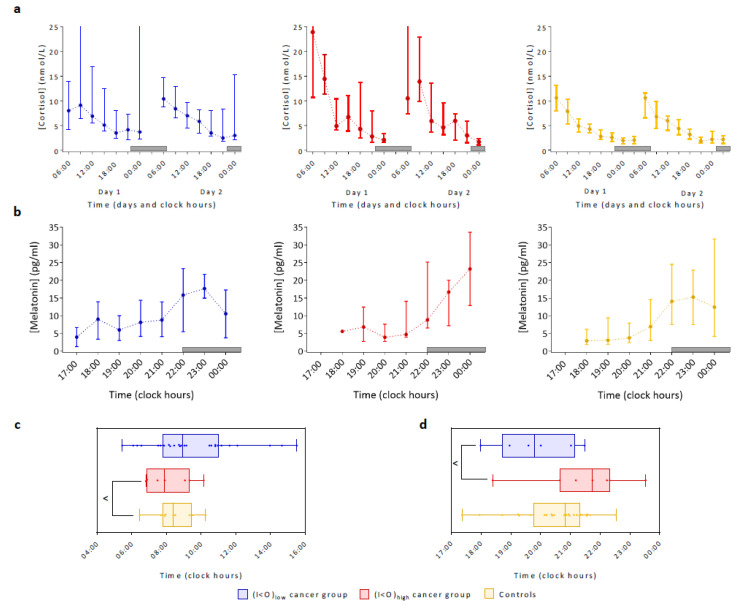
Daily patterns in salivary cortisol and melatonin concentrations in cancer patients and in controls. (**a**) Circadian changes in salivary cortisol over two consecutive days in the (I < O)_low_ patients (blue), in the (I < O)_high_ patients (red) and in the controls (yellow). (**b**) Hourly changes in evening salivary melatonin concentrations in the (I < O)_low_ patients (blue), in the (I < O)_high_ patients (red), and in the controls (yellow). (**c**) Box plots of cortisol circadian acrophase in both I < O patients’ groups and in controls. (**d**) Box plots of dim light melatonin onset (DLMO) in both I < O patients’ groups and in controls. In (**a**,**b**), bars represent the interquartile ranges and dots plot the median values. The grey bar on the abscissa represents the night span (22:01 to 07:00). Levels of significance: ^ 0.05< *p* ≤0.1 (two-tailed Welch’s modified *t*-test).

Salivary cortisol time series exhibited consistent diurnal changes from early morning “highs” to evening “lows” over two consecutive days in most individual controls and cancer patients, irrespective of their I < O category (Figure 6a).

Inter-subject variability was markedly larger for cancer patients compared to controls. A circadian rhythm was estimated with a cosinor modelling approach for 16 out of 22 cancer patients (73%), including nine out of 13 with (I < O)_low_ (69%), and seven out of nine (78%) with (I < O)_high_, and for 26 out of 30 controls (87%).

The rate of subjects with a significant cortisol rhythm was similar between the (I < O)_high_ patients and controls (*p* = 0.70). No significant difference between both I < O groups was found for any of the cortisol circadian parameters (Figure 6c). Surprisingly, the cortisol amplitude in the (I < O)_high_ group was larger than that of controls (*p* = 0.01) but neither mesor nor acrophase differed significantly between both groups.

The median salivary melatonin concentrations increased over time from 18:00 to 23:00 both in cancer patients and in controls, with large between-subject variations in each group (Figure 6b). Baseline melatonin levels were higher in cancer patients than in controls. The evening rise in salivary melatonin levels from baseline was three-fold in cancer patients with (I < O)_low_, five-fold in those with (I < O)_high_, and six-fold in controls. Twenty patients (80%) and 30 controls (100%) provided sufficient melatonin samples for dim light melatonin onset (DLMO) computation. However, individual baselines of salivary melatonin could only be identified for six patients (24%) and 12 controls (40%), while the estimation for a further seven patients and 12 controls was based on averaged data from the individual baseline profiles. Patients in the (I < O)_low_ group tended to have an earlier DLMO than those in the (I < O)_high_ group (19:48 vs. 21:44, *p* = 0.08). The median DLMO time was at 20:28 in controls (Figure 6d).

### 2.5. Sleep

**Figure 7:**
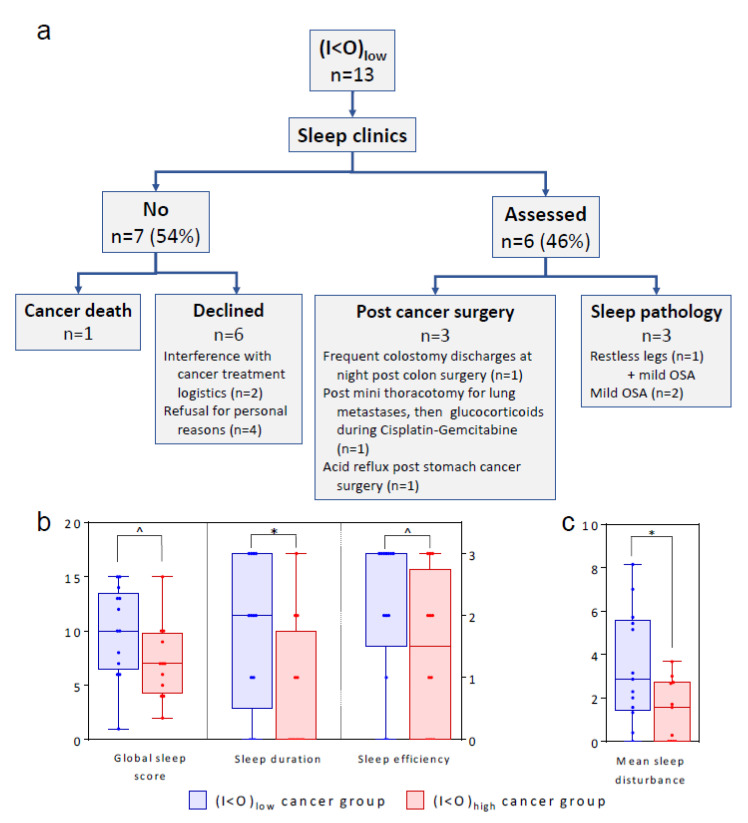
Sleep pathology assessment results and subjective sleep parameters. (**a**) Consort diagram of the sleep clinics assessment. OSA stands for obstructive sleep apnea. (**b**) Box plots showing distribution of global score and two selected components of the Pittsburgh sleep quality index (PSQI) in both I < O groups. (**c**) Box plots of the mean score of the sleep disturbance symptom from the MDASI questionnaire in both I < O groups. In (**b**,**c**), bars represent the extremes and bold lines inside the box plots plot median levels. Values for each individual are plotted as jittered dots to better show the distribution. Levels of significance: * 0.01 < *p* ≤ 0.05; ^ 0.05 < *p* ≤ 0.1 (two-tailed Welch’s modified *t*-test).

Six out of the 13 (I < O)_low_ patients (46%) were diagnosed with insomnia following their sleep clinics attendance: three due to post-cancer surgery complications, and three with confirmed mild obstructive sleep apnea (OSA) with an apnea-hypopnea index (AHI) of 9.7, 10.9, and 7.9 respectively. One of the OSA patients also suffered from periodic leg movements (Figure 7a). The subjective sleep data revealed a trend towards more sleep problems in the (I < O)_low_ group (Figure 7b,c), considering global sleep score (*p* = 0.08), subjective sleep duration score (*p* = 0.046) in PSQI, and average sleep disturbance score in the M.D. Anderson Symptom Inventory (MDASI) questionnaire (*p* = 0.024).

In controls, none of whom reported any sleep trouble, the subjective evaluation of sleep using a different 10-points scale showed that their sleep quality was rather good, as revealed by an average score of 7.3 ± 1.5 A.U.

### 2.6. Physical Activity

Two of the (I < O)_low_ patients referred to a physiotherapist displayed signs of physical pathologies, including restless legs (confirmation from sleep clinics), fatigue, and breathlessness. In their daily diaries, ten out of the 11 (I < O)_high_ patients (91%) reported daily physical exercise activities of 30 min or more over at least four study days, as compared to two out of the 13 (I < O)_low_ patients (15%) (*p* = 0.001), as well as to 17 of 33 controls (51%) (*p* = 0.03).

### 2.7. Relevance of Age, Sex and Cancer on Circadian Parameters

In controls, age, but not sex, significantly influenced the four main circadian parameters (Supplementary Materials Table S1). Participants aged 40 years or over had reduced I < O and P1-1 (*p* = 0.01 and *p* = 0.0009, respectively) as well as phase-advanced circadian activity acrophase and center-of-rest time (*p* = 0.01 for both).

Since all cancer patients were 40 years or older, we further compared the circadian and sleep parameters of the (I < O)_high_ cancer patients to the older controls (Appendix B, Table S2). We found that their I < O values were similar (median, 97.3% vs. 98.3%, respectively; *p* = 0.15). The majority of the other rest-activity and temperature circadian parameters were also similar although the (I < O)_high_ cancer patients were less active, and had more sleep disturbances, as shown by differences in activity level and P1-1 (*p* < 0.0001 and *p* = 0.01, respectively).

### 2.8. Regression Analysis to Identify Main Actionable Determinants of (I < O)

The domains and factors that significantly influenced the I < O in cancer patients using pairwise correlations (*r*) were:

1. The day-to-day variability in sleep duration estimated with hidden Markov modelling (HMM) (*r* = −0.53, *p* = 0.009);
2. The self-reported exercise (*r* = 0.48, *p* = 0.02), the rest-activity circadian amplitude (*r* = 0.73, *p* < 0.0001), the median activity out-of-bed (*r* = 0.68, *p* = 0.0003), and the level of activity (*r* = 0.56, *p* = 0.005);
3. The day-to-day variability in the self-reported retiring time (*r* = 0.49, *p* = 0.02);
4. The physiologic chest temperature rhythm (24-h dominant period and nocturnal acrophase between 22:01 and 07:00, *p* = 0.03);
5. The chronotype score (*r* = −0.43, *p* = 0.04).

Both the circadian amplitude in rest-activity and sleep duration variability were identified as best predictors of a patient’s I < O, according to multiple linear regression and statistical selection procedures, using parameters from 7-day chest rest-activity time series. The estimated regression equation (Equation (1)), where E(I < O ) stands for estimated I < O, was:

E(I < O) = 0.1584 * chest activity amplitude (*p* < 0.0001) − 2.6695 * sleep duration variability (*p* = 0.0001) (1)

where the (adjusted) R-squared of the regression was 0.7869 for the sample size of 22 patients. The same covariates also significantly influenced the I < O in the controls, i.e.:

1. The HMM-estimated sleep duration variability (*r* = −0.53, *p* = 0.002);
2. The rest-activity circadian amplitude (*r* = 0.36, *p* = 0.04);
  but in addition age (*r* = −0.48, *p* = 0.006).

In the controls, statistical procedures from multiple regression fitting models also selected rest-activity rhythm amplitude and sleep duration variability as the best predictors of the I < O, together with age. The estimated regression equation (Equation (2)) was as follows:

E(I < O) = −1.300244 * sleep duration variability (*p* = 0.0001) + 0.020682 * activity amplitude (*p* = 0.04) − 0.035849 * age (*p* = 0.0001) (2)

with an adjusted R-squared of 0.5858 in 32 controls.

## 3. Discussion

A strong engagement by 25 patients with advanced gastro-intestinal cancer and 33 controls provided a multidimensional data set over one week, with median total protocol compliance rates exceeding 95%. Results from studies involving wearable sensors and regular patient reported outcome measure (PROM) assessment, have highlighted the paramount importance of the engagement of both patients and health professionals in order to improve outcomes [26,27]. Both studies provided excellent quality data for activity and temperature from the tele-transmitting chest sensor and for the questionnaires completed at home. Neither participants nor carers raised concerns regarding the equipment, and no adverse feelings in the participants’ experience were documented. The compliance figures with PROMs in cancer patients were better than those found using the inCASA fixed platform, and compared favorably with other studies using daily mHealth symptoms checklists or even less frequent digital symptom assessment methods [28,29,30]. While salivary cortisol data were of high quality for almost all participants, melatonin secretion mechanisms might be impaired in cancer patients and in controls older than 40 y.o., for whom DLMO could hardly be precisely computed in real life conditions [31]. Beta-blockers intake could indeed contribute to the lack of technical determination of DLMO for three of four patients on such medications, but played no role for the other patients nor for controls. The intake of paracetamol or non-steroidal anti-inflammatory drugs had no apparent effect on the chest surface temperature patterns of cancer patients. Thus, five of six patients on either drug displayed a circadian rhythm, with an acrophase located at night for four of them, i.e., similar to the results in the controls.

As anticipated and consistent with prior reports, we observed a nearly 50:50 percentage split of patients with a good and a poor rest-activity rhythm (threshold for I < O of 97.5) [10,11,12,13]. The circadian rhythm in rest-activity was not significantly influenced by patients’ demographics, cancer characteristics, environmental synchronization signals, or lifestyle. Nevertheless, the I < O index was strongly and consistently correlated with other rest-activity rhythm parameters, each addressing a different perspective on the rest-activity circadian domain. Moreover, the rest-activity rhythm parameters were significantly associated with the occurrence of a physiologic 24-h rhythm in chest surface temperature, involving an acrophase at night. We identified in this study relevant associations between I < O and physical activity parameters, subjective and objective, as well as subjective sleep quality and objective sleep duration and timing. The most influential factors on I < O in both cancer patients and controls were the indicators in the physical activity domain and the variability in duration in the sleep domain. Some previously undiagnosed sleep disorders that deserve further attention were identified in the cancer patients.

The study in cancer patients (IDEAs) has highlighted that low I < O values were associated with disrupted circadian coordination, poor physical activity and irregular daily living activities in cancer patients. Based on the evidence collected here for (I < O)_low_ patients without sleep pathology, timed physical exercise during the day, regular times of retiring and meals, and a low dose of (modified-release) hydrocortisone (in case of associated (I < O)_low_ and low r24) could be recommended [32,33,34,35].

## 4. Materials and Methods

### 4.1. Study Designs and Participants

**Figure 8:**
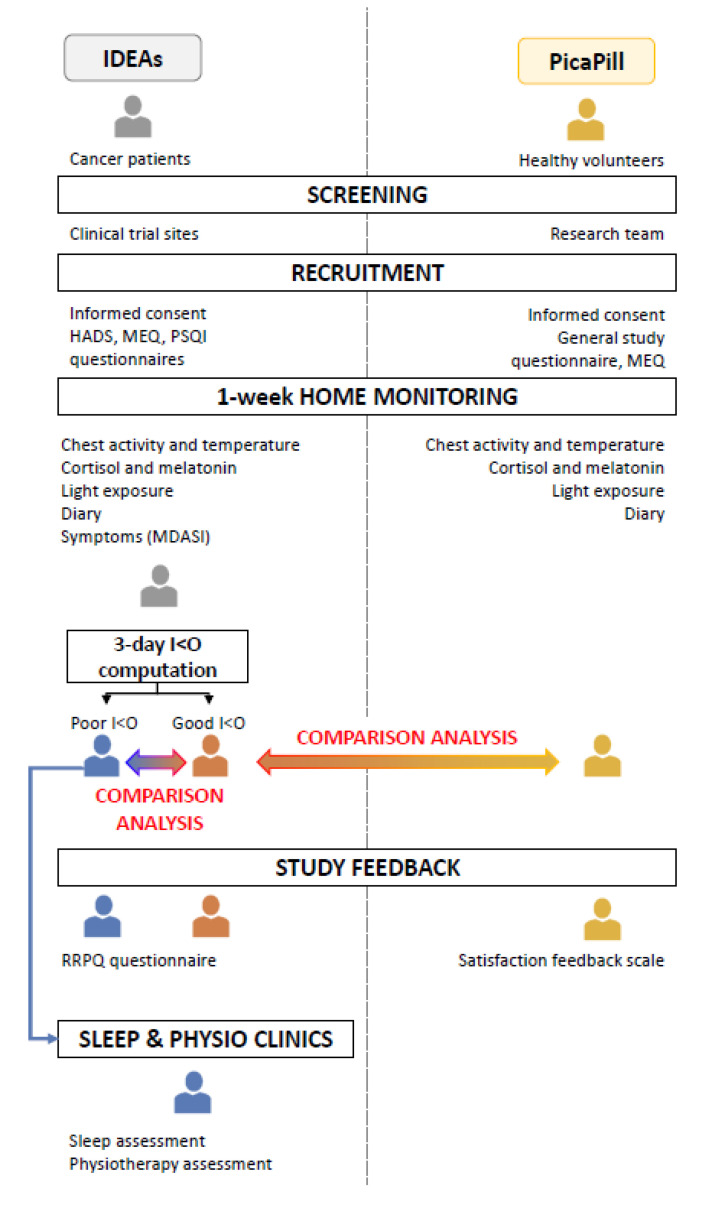
Study design. Chart showing the similar study designs for cancer patients (IDEAs) and controls (PicaPill). HADS: hospital anxiety and depression scale; MEQ: Morningness-Eveningness Questionnaire; PSQI: Pittsburgh sleep quality index; MDASI: M.D. Anderson Symptom Inventory; RRPQ: reactions to research participation questionnaire.

Two studies, in the sequel named “IDEAs” and “PicaPill”, were subject to a similar design, facilitating comparison between cancerous and control participants (Figure 8).

Written informed consent was received from all participants prior to inclusion, and the studies were conducted according to the Helsinki Declaration [36]. For IDEAs, patients with locally advanced or metastatic gastro-intestinal cancer and a good performance status were enrolled at five United Kingdom hospitals, before they received a new chemotherapy protocol. The PicaPill study aimed to identify the influence of age and sex on circadian biomarkers in stratified control participants, and proposed a novel model for the estimation of the internal circadian phase [37]. The adult volunteers in both studies had their chronotype determined using the MEQ [38]. They continuously wore a thoracic sensor containing an accelerometer and skin surface thermometer (Movisens, Karlsruhe, Germany), as well as a wrist-watch with actigraph and luminometer (Motionlogger MicroWatch, Ambulatory Monitoring Inc., Ardsley, NY, USA) and maintained a lifestyle diary for 7 days. Anonymized data from the thoracic sensor were automatically tele-transmitted via GPRS daily. Participants were also asked to provide: (i) 12 saliva samples to be collected every 3 h in the daytime for 2 days, for the determination of diurnal salivary cortisol patterns; and (ii) 6 saliva samples gathered every hour starting at 19:00 and in dim light, for the DLMO computation.

The cancer patients also completed a PSQI, a HADS, and a reaction to research participation questionnaire (RRPQ) [39,40,41]. They rated their symptoms according to the MDASI at home each evening for 7 days [42].

### 4.2. Data Collection and Management

Tele-transmitted chest sensor data were stored on the server based on HL7 standards (international standards for transfer of clinical and administrative data). Anonymized data were saved on a secure storage server according to the national Data Protection and Freedom of Information Acts guidance.

Data transmission was inspected at least twice a week during the monitoring sessions to ensure adequate data collection. Missing values (typically sensor for a shower or a bath to avoid contact with water) were identified through steady decreases in temperature measures down to room temperature values, and were removed from analyses. Light intensity exposure was measured every minute using the wrist actigraph, worn continuously for 7 days. The data from patients were downloaded at the end of the study by the research nurse at the investigational site. The anonymized data file was emailed to the study team. The data from controls were handled directly by the study team. We report here the light exposure data, because of its importance for the synchronization of circadian rhythms and for the interpretation of melatonin secretion patterns.

All salivary samples were stored in the fridge or freezer at the participants’ home, and returned at end of study participation to the investigational site for storing at −80 °C until hormonal determinations (Surreys Assays LTD, Guildford, Surrey, UK).

The study team accessed the anonymized data on the server. Missing values of chest actigraph and temperature records occurred when the participant removed the sensor, typically for a shower or a bath to avoid contact with water. The missing values were identified through steady decreases in temperature measures down to room temperature values and were removed from analyses.

The tele-transmitted data were analyzed in real time after an initial recording span of 72 h, such that patients whose I < O after 72 h ((I < O)_72h_) was lower than 97.5% were referred to sleep and physiotherapy clinics, including polysomnography or electroencephalography whenever medically-indicated. Obstructive sleep apnea was classified according to the AHI [43].

### 4.3. Statistical Methods

Two-tailed Welch’s *t*-tests for continuous variables and Fisher’s exact test for categorical variables were used for pairwise comparisons. To identify relations between variables, Spearman correlations were considered. Significance was defined as *p*-value < 0.05 and possible trends were considered for 0.05 < *p* < 0.15.

#### 4.3.1. Circadian Parameters

The I < O index is defined as the percentage of activity counts per minute recorded when the subject is in-bed at night, with values lower than the median activity count when the subject is out-of-bed during the daytime. The I < O value of cancer patients was first computed using the first 72 h of recordings from the chest sensor ((I < O)_72h_), so as to categorize them in the (I < O)_high_ group if (I < O)_72h_ was above 97.5% or in the (I < O)_low_ group if (I < O)_72h_ was below or equal to 97.5%, which led to referral to sleep and physiotherapy clinics. I < O was also computed at the end of the study over the 7 days of chest activity recordings for all 58 participants

A hidden Markov model was fitted to 5-min aggregated chest rest-activity data, which retrospectively infers the times an individual spent in three different states, that we will define as an inactive/rest (IA), moderately active (MA), and highly active (HA) state, and are specific to the person [25]. The HMM further produces a variety of numerical quantifiers that are of interest to circadian rhythm in rest-activity, such as:

1. The mid values of the MA and HA states which indicate daily activity levels;
2. The rhythm index (RI), with values ranging between 1, corresponding to best average quality and regularity of the IA state, and 0, corresponding to poor quality and absence of a consistent rest state in the pattern;
3. The average center-of-rest time of the IA state;
4. P1-1, the estimated probability of staying in the IA state (state 1); when having previously been in this state. P1-1 is positively correlated with I < O, while the probability [1-(P1-1)] serves as an estimate of rest interruption.

The day-to-day sleep onset, wake up time and length of rest were also estimated via the HMM approach.

In addition, the autocorrelation coefficient at 24-h delay (r24) was computed to characterize the regular overall circadian regularity in rest-activity pattern, with possible values ranging from −1 to +1. The range of interest usually lies between values around zero for no regularity, and rising values up to 1 indicating a more daily recurring pattern in activity [13].

#### 4.3.2. Spectral Analysis and Cosinor Modelling

Spectral analysis combined with spectrum-resampling methods was applied to hourly aggregated chest rest-activity, surface temperature, and light data, after missing data were linearly interpolated [24]. Period length was estimated by the dominant spectral period and corresponding amplitude and acrophase (time of estimated maximum) along with 90% confidence intervals.

The surface temperature rhythm was fitted by a cosinor model (Equation (3)). Data were first smoothed by a 1-h moving average window, then computed as an averaged 24-h profile y(t), and fitted using a two-harmonic cosinor model, with periods $T_{1}=24\;h$ and $T_{2}=12$ h:

(3) $$y\left(t\right)=M+a_{1}\sin\left(\frac{2\pi t}{T_{1}}\right)+b_{1}\cos\left(\frac{2\pi t}{T_{1}}\right)+a_{2}\sin\left(\frac{2\pi t}{T_{2}}\right)+b_{2}\cos\left(\frac{2\pi t}{T_{2}}\right)+e\left(t\right)$$

where y(t) is the temperature at time *t*, M is the mesor (mean level of the fitted cosine function), $a_{1},a_{2}$ and $b_{1},b_{2}$ are the coefficients of the cosinor model, and e(t) is the error [44]. Given the periods $T_{1}$ and $T_{2}$, the coefficients were estimated by least-squares linear regression.

Cortisol time series were considered as adequate if at least 9 samples were collected over 2 days. A cosinor model with a period of 24-h was fitted to describe the circadian pattern of salivary cortisol secretion (Equation (4)):

(4) $$y\left(t\right)=M+a\sin\left(\frac{2\pi t}{T}\right)+b\cos\left(\frac{2\pi t}{T}\right)+e\left(t\right)$$

where y(t) is the salivary cortisol secretion at time *t*; M is the mesor; a and *b* are real coefficients; e(t) is the error term. Given the period T=24 h, y(t) was fitted by a linear regression model. We computed the cortisol mesor, amplitude ($\sqrt{a^{2}+b^{2}}$), and acrophase (time of estimated maximum in fitted $\hat{y}$), with 90% confidence intervals, estimated by the bootstrap method [45].

#### 4.3.3. DLMO Computation

The dim light conditions required for the DLMO determinations were checked by computing the average level of light and duration of exposure over the 30 min before each saliva sample collection.

DLMO is commonly computed as the time of the day when melatonin concentration in plasma or saliva exceeds a threshold computed as the mean of 3 consecutive daytime values before melatonin rise plus twice the standard deviation of these 3 points [31]. For those participants with insufficient baseline data, an estimated threshold value was computed as the mean plus twice the standard deviation of the pooled baseline melatonin values in the participants with adequate baseline data.

This estimated threshold was first validated in the participants with adequate baseline data by Pearson’s correlation, before its use for all the participants. Distinct estimated thresholds were computed for the patients and the controls.

#### 4.3.4. Analysis of RACR Domain

The rest activity circadian rhythm (RACR) domain was described by the (I < O)_72h_ value estimated after the initial 72 h and categorized as (I < O)_low_ versus (I < O)_high_, and the four circadian parameters (I < O, P1-1, RI, and r24) computed over 7 days, each describing different aspects of the RACR domain.

Potentially modifiable variables influencing RACR were considered in six functional domains: sleep, physical activity, lifestyle, psychosocial, ‘other biomarkers’ (chest temperature and salivary cortisol), and symptoms. Non-modifiable factors such as patient’s characteristics and cancer characteristics were also examined. Pairwise comparisons were performed to identify adequate variables to use as covariates in regression analysis. Regression analysis was applied by considering the five RACR parameters as dependent variables and the modifiable, as well as non-modifiable variables as potential predictors. A regression model was identified by the global model selection based on the (corrected) Akaike’s information criterion (AICc) using the R package MuMIn. Here, we focus on results for I < O.

### 4.4. Study Approval

The Research Ethics Committees, Health Research Authority, East Midlands—Leicester Central Research Ethics Committee approved the IDEAs study (IRAS 233972). The PicaPill study was approved by the Ethical Committee of Warwick University (REGO-2017-2055). For both studies, a written informed consent was received from all participants prior to inclusion.

## 5. Conclusions

In conclusion, we found that the longitudinal collection of tele-transmitted rest-activity and body temperature during daily routine for one week were sufficient for the identification of precise and potentially actionable factors for personalized interventions, aiming to improve circadian and sleep alterations. Our study promotes a patient-centered approach based on e-Health platforms and patient engagement. Such a framework challenges the current cancer patient pathways. Indeed, a multi-professional healthcare coordination structure is pivotal for the success of such projects, necessitating a shift in current hospital-centered health care.

## Figures and Tables

**Table 1:** Participants’ main characteristics. When not specified the data are presented as Number of subjects (%).

Number of Participants	Cancer Patients 25	(I < O)_low_ Cancer Group 13	(I < O)_high_ Cancer Group 12	Controls 33	*p*-Values ^1^	
					(I < O)_low_ vs. (I < O)_high_ (Cancer)	(I < O)_high_ (Cancer) vs. Controls
**Sex**						
M	21 (84.0)	11 (84.6)	10 (83.3)	15 (45.5)	1	0.04 *
F	4 (16.0)	2 (15.4)	2 (16.7)	18 (54.5)		
**Age (years)**						
Median	66	66	70	35	0.568	<0.0001 ****
Range	40–82	40–80	42–82	21–78		
**BMI**						
Median	27.5	27.5	26.9	24.4	0.511	0.216
Range	19.8–38.8	22.4–38.8	19.8–33.7	18.9–42.0		
**(I < O)_72h_ (%)**						
Median	97.4	90.4	98.7	NA	0.0017 **	NA
Range	67.6–100	67.6–97.4	97.7–100	NA		
**Work status**						
Employed or self-employed	8 (32.0)	5 (38.5)	3 (25.0)	15 (45.5)	0.673	0.002 **
Student	0 (0)	0 (0)	0 (0)	11 (33.3)		
Retired or not working	17 (68.0)	8 (61.5)	9 (75.0)	7 (21.2)		
**Chronotype**						
Definite morning	3 (12)	1 (8)	2 (17)	5 (15.2)	0.408	0.594
Moderate morning	12 (48)	8 (62)	4 (33)	10 (30.3)		
Intermediate	9 (36)	4 (31)	5 (42)	15 (45.5)		
Moderate evening	0 (0)	0 (0)	0 (0)	3 (9.1)		
Not available	1 (4)	0 (0)	1 (8)	0 (0)		
**Ongoing medical condition (other than cancer)**						
None	8 (32.0)	3 (23.1)	5 (41.7)	25 (75.8)	0.362	0.003 **
1–2	8 (32.0)	6 (46.2)	2 (16.7)	7 (21.2)		
≥ 3	9 (36.0)	4 (30.8)	5 (41.7)	1 (3.0)		
**Concurrent medications (apart from cancer treatments)**						
0	3 (12.0)	1 (7.7)	2 (16.7)	25 (75.8)	0.866	< 0.0001 ****
1–2	12 (48.0)	7 (53.8)	5 (41.7)	8 (24.2)		
≥ 3	10 (40.0)	5 (38.5)	5 (41.7)	0 (0)		
**Site of primary tumor**						
Colorectal	14 (56.0)	5 (38.5)	9 (75.0)	NA	0.111	NA
Other	11 (44.0)	8 (61.5)	3 (25.0)	NA		
**Cancer status**						
No residual tumor	2 (8.0)	2 (15.4)	0 (0)	NA	0.561	NA
Locally advanced cancer	6 (24.0)	3 (23.1)	3 (25.0)	NA		
Metastatic disease	17 (68.0)	8 (61.5)	9 (75.0)	NA		
**Number of metastatic sites**						
0	8 (32.0)	5	3	NA	0.861	NA
1–2	13 (52.0)	6	7	NA		
≥ 3	4 (16.0)	2	2	NA		
**Main metastatic sites**						
Liver	9 (36.0)	3 (23.1)	6 (50.0)	NA	0.721	NA
Lymph nodes	9 (36.0)	5 (38.5)	4 (33.3)	NA		
Lungs	8 (32.0)	5 (38.5)	3 (25.0)	NA		
Other	5 (20.0)	2 (15.4)	3 (25.0)	NA		
**Prior cancer treatments**						
Surgery	13 (52.0)	6 (46.1)	7 (58.3)	NA	0.508	NA
Radiotherapy	8 (32.0)	2 (15.4)	6 (50.0)	NA		
Chemotherapy	20 (80.0)	10 (76.9)	10 (83.3)	NA		

Abbreviation: NA: not applicable, ^1^ Differences between the (I < O)_high_ and the (I < O)_low_ cancer patients and between the cancer patients and controls were tested with two-tailed Welch’s *t*-tests for continuous variables or Fisher’s exact test for categorical variables. The corresponding *p*-values are shown as follows: **** *p* < 0.0001; ** 0.001 < *p* ≤ 0.01; * 0.01 < *p* ≤ 0.05.

## Supplementary Materials

The following are available online at https://www.mdpi.com/2072-6694/12/7/1938/s1, Figure S1: Environmental synchronizing signals. Median, interquartiles, and individual median timings of each environmental signal according to either I < O cancer group (11 to 13 participants for each signal) or control group (31 to 33 participants), Table S1: Sex or age effects on the rest-activity and chest surface temperature circadian parameters, synchronization signals, cortisol rhythm, and DLMO in the controls, Table S2: Comparison of rest-activity and chest surface temperature circadian parameters, synchronization signals, cortisol rhythm and DLMO between the (I < O)_high_ patients and the controls older than 40 years.

## Appendix A

We wondered if the differences, found in circadian biomarkers parameters between the (I < O)_high_ or (I < O)_low_ patients and the controls, could reflect differences in the recurring environmental signals that synchronize the endogenous circadian rhythms. We addressed this question by analyzing the precise daily diaries that were provided by 24 out of 25 cancer patients and 32 out of 33 controls.

Again, substantial inter-subject differences were apparent regarding the daily-recurrent environmental signals, in both cancer patients and controls (Figure S1). Differences were neither found between the cancer patients with (I < O)_low_ or (I < O)_high_, nor with controls, regarding the times of retiring and estimated sleep onset (p > 0.40), the times of breakfast, lunch, or dinner (*p* > 0.07), or the acrophase of light exposure (*p* = 0.34). In contrast, however, the (I < O)_high_ cancer patients took dinner and retired earlier than the controls (*p* = 0.003 and *p* = 0.03, respectively). The fitted HMM further revealed that they also woke up later (*p* = 0.02) and went to sleep earlier (*p* = 0.02) than the controls.

While the times of awakening and sleep onset were found to be significantly influential on the I<O in cancer patients, this was not the case for the same parameters recorded in the diaries. We therefore concluded that the daily diary reports, despite their careful annotations, provided no information regarding circadian and sleep cycles that was going beyond the analyses of the tele-transmitted activity and temperature sensor data. Interestingly, no significant correlation was found between the subject’s chronotype and any lifestyle or timing parameter based on the circadian biomarkers. This indeed suggested that the adjustment of the central circadian coordination system to the environmental synchronizer varied between subjects irrespective of whether they had cancer or not.

## Appendix B

The phase advance in the older control participants was supported by self-reported and tele-monitored wake-up times (*p* = 0.01 and *p* = 0.01 respectively), self-reported retiring times (*p* = 0.02) and tele-monitored sleep onset (*p* = 0.01). We could not identify notable effects of age or sex regarding other variables or parameters, namely level of activity, circadian amplitude, r24, RI, and rest duration, as well as synchronization signals, such as light exposure, times of awakening or retiring, and meal times.

Regarding chest surface temperature, older participants displayed a larger circadian amplitude (spectral analysis, *p* = 0.04; cosinor, *p* = 0.05). Females had a higher temperature mesor (*p* = 0.0002) and tended to display a later circadian acrophase (*p* = 0.07).

Neither self-reported sleep quality nor salivary cortisol rhythm parameters varied with age (*p* = 0.90) or sex (*p* = 0.93). Interestingly, the melatonin rise was significantly steeper in the controls who were younger than 40 years, while the average pattern of the older subjects was similar to that of the cancer patients. An individual melatonin baseline was identified for 10 of the 15 controls aged less than 40 years (67%), as compared to only 2 of the 15 older controls (13%) (*p* = 0.01). The DLMO times did not vary according to age, but they occurred earlier in females (*p* = 0.04).

The (I < O)_high_ cancer patients also had a larger circadian cortisol amplitude (*p* = 0.01), rose later in the morning (*p* = 0.01), and had dinner earlier (*p* = 0.02).
