# Supplementary material for: Tele-Monitoring of Cancer Patients’ Rhythms during Daily Life Identifies Actionable Determinants of Circadian and Sleep Disruption
Source: Cancers (Basel). 2020 Jul 17;12(7):1938. doi: 10.3390/cancers12071938 (PMC7409071; doi:10.3390/cancers12071938)
Supplement: Supplementary file 1 [file cancers-12-01938-s001.pdf]

cancers

MDPI

Article

Tele-Monitoring of Cancer Patients' Rhythms during Daily Life Identifies Actionable Determinants of Circadian and Sleep Disruption.

Francis Lévi [1,2,3,*], Sandra Komarzynski [1,2], Qi Huang [1,4], Teresa Young [5], Yeng Ang [6,7], Claire Fuller [8], Matei Bolborea [1], Julia Brettschneider [4], Joanna Fursse [1], Bärbel Finkenstädt [2,4], David Pollard White [9,10] and Pasquale Innominato [1,2,8]

[1] Cancer Chronotherapy Team, Warwick Medical School, Coventry CV4 7AL, UK; S.Komarzynski@warwick.ac.uk (S.K.); Q.Huang.6@warwick.ac.uk (Q.H.); M.Bolborea@warwick.ac.uk (M.B.); Joanna.fursse@nhs.net (J.F.); Pasquale.Innominato@wales.nhs.uk (P.I.)
[2] European Laboratory U935, Institut National de la Santé et de la Recherche Médicale (INSERM), and Paris-Saclay University, Villejuif 94801, France; B.F.Finkenstadt@warwick.ac.uk
[3] Hepato-biliary Centre, Paul Brousse Hospital, Assistance Publique Hôpitaux de Paris (AP-HP), Villejuif 94800, France
[4] Department of Statistics, University of Warwick, Coventry CV4 7AL, UK; Julia.Brettschneider@warwick.ac.uk
[5] Mount Vernon Cancer Centre, East and North Hertfordshire NHS Trust, Northwood, Middlesex HA6 2RN, UK; Teresa.young2@nhs.net
[6] Salford Royal NHS Foundation Trust, Salford M6 8HD, UK; Yeng.Ang@srft.nhs.uk
[7] Gastrointestinal Sciences, Division of Diabetes, Endocrinology and Gastroenterology, Faculty of Biology, Medicine and Health, University of Manchester, Manchester M13 9PL, UK
[8] North Wales Cancer Treatment Centre, Ysbyty Gwynedd, Betsi Cadwaladr University Health Board, Bangor LL57 2PW, UK; Claire.Fuller2@wales.nhs.uk
[9] Division of Sleep and Circadian Disorders, Brigham and Women's Hospital, Harvard Medical School, Boston, MA 02130, USA; dpwhite@partners.org
[10] Philips Respironics, Murrysville, PA 15668, USA
* Correspondence: F.Levi@warwick.ac.uk; Tel.: +44-2476-575-132

Supplementary

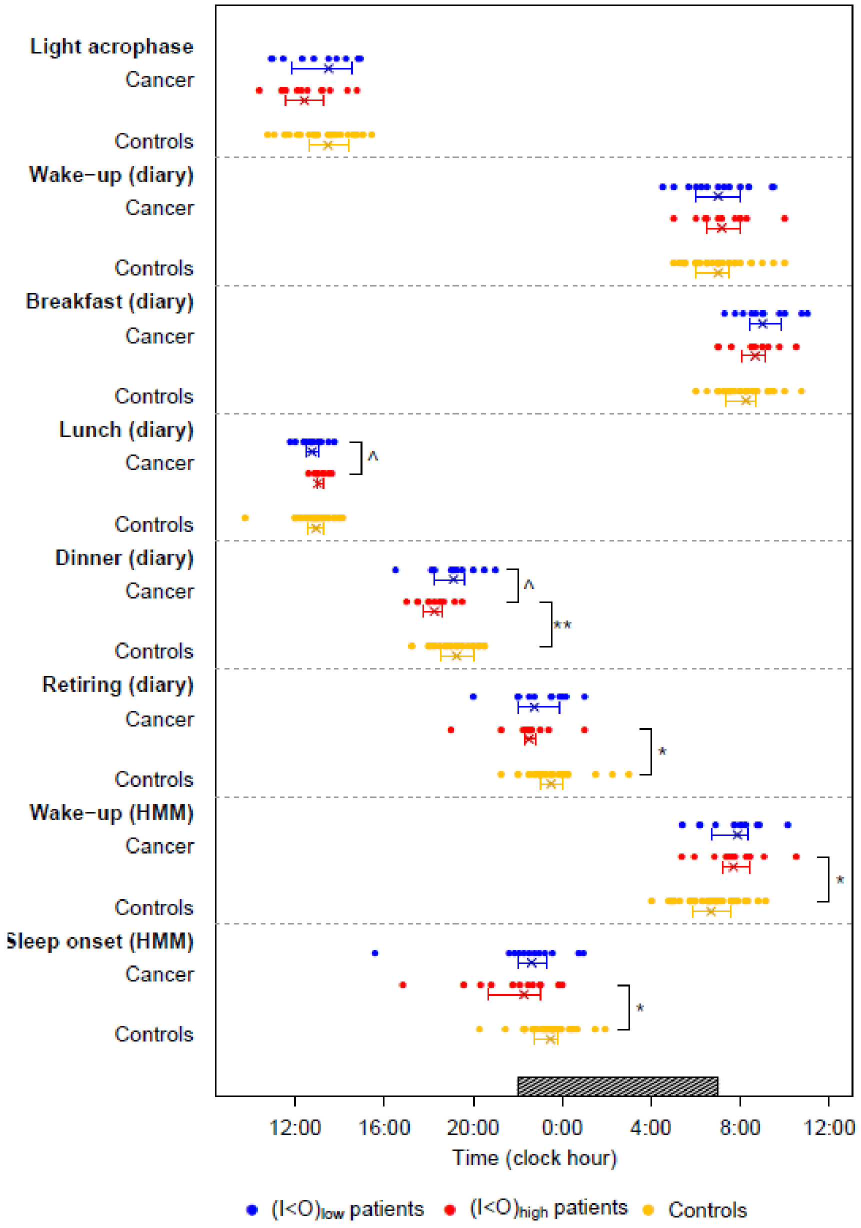

Figure S1. Environmental synchronizing signals. Median, interquartiles, and individual median timings of each environmental signal according to either I < O cancer group (11 to 13 participants for each signal) or control group (31 to 33 participants).

cancers

MDPI

Table S1. Sex or age effects on the rest-activity and chest surface temperature circadian parameters, synchronization signals, cortisol rhythm, and DLMO in the controls. When not specified the data are presented as Number of subjects (%).

	**Age < 40 years**	**Age ≥ 40 years**	**Male**	**Female**
**Rest-activity circadian rhythm**	**N=18**	**N=15**	**N=15**	**N=18**
I<O over study week (%)	p=0.01*		p=0.98	
Median [Q1, Q3]	99.7 [98.9, 100.0]	98.3 [97.5, 99.4]	99.2 [98.4, 99.8]	99.1 [98.4, 99.8]
Range	98.0 - 100.0	95.0 - 100.0	95.0 - 100.0	95.5 - 100.0
Median of moderate-to-high activity (acc/min)	p=0.22		p=0.41	
Median [Q1, Q3]	172.7 [162.5, 185.8]	185.3 [172.9, 199.6]	174.7 [158.1, 194.4]	178.6 [169.2, 190.9]
Range	132.5 - 208.5	142.7 - 210.0	132.5 - 208.5	149.7 - 210.0
Circadian amplitude (acc/min)	p=0.17		p=0.27	
Median [Q1, Q3]	61.1 [53.1, 69.1]	67.5 [63.0, 78.1]	62.5 [54.8, 80.1]	66.5 [54.6, 71.4]
Range	39.3 - 105.2	39.5 - 101.5	45.6 - 105.2	39.3 - 82.2
Circadian acrophase (clock time)	p=0.01*		p=0.71	
Median [Q1, Q3]	15:07 [14:11, 16:14]	14:00 [12:29, 14:50]	14:33 [12:48, 15:23]	14:31 [13:57, 15:50]
Range	11:59 - 17:10	11:43 - 16:01	11:59 - 17:10	11:43 - 16:49
P1-1	p=0.0009***		p=0.63	
Median [Q1, Q3]	0.97 [0.97, 0.98]	0.96 [0.94, 0.97]	0.97 [0.96, 0.98]	0.97 [0.96, 0.97]
Range	0.96 - 0.99	0.93 - 0.98	0.94 - 0.99	0.93 - 0.98
r24	p=0.30		p=0.39	
Median [Q1, Q3]	0.36 [0.30, 0.41]	0.39 [0.32, 0.45]	0.39 [0.29, 0.42]	0.34 [0.33, 0.44]
Range	0.09 - 0.63	0.20 - 0.55	0.17 - 0.63	0.09 - 0.47
Rhythm Index	p=0.23		p=0.21	
Median [Q1, Q3]	0.79 [0.74, 0.83]	0.74 [0.58, 0.80]	0.79 [0.74, 0.84]	0.77 [0.63, 0.81]
Range	0.47 - 0.90	0.48 - 0.92	0.57 - 0.92	0.47 - 0.90
Rest duration (hours)	p=0.56		p=0.85	
Median [Q1, Q3]	8.0 [7.4, 8.8]	8.3 [7.5, 9.4]	8.1 [7.7, 8.9]	7.9 [7.3, 8.9]
Range	7.1 - 12.4	5.3 - 12.9	6.8 - 12.9	5.3 - 12.4

Center-of-rest time (clock time)	p=0.01*		p=0.96	
Median [Q1, Q3]	03:22 [02:41, 03:47]	02:20 [01:48, 03:08]	03:00 [01:52, 03:22]	03:08 [02:24, 03:29]
Range	01:20 - 06:25	01:10 - 03:50	01:10 - 06:25	01:20 - 05:20
**Chest surface temperature circadian rhythm**	**N=9**	**N=12**	**N=10**	**N=11**
Circadian amplitude (°C) - SR	p=0.04*		p=0.37	
Median [Q1, Q3]	0.7 [0.5, 0.8]	1.1 [0.6, 1.3]	1.0 [0.7, 1.3]	0.7 [0.5, 0.9]
Range	0.4 - 1.0	0.2 - 2.1	0.5 - 1.5	0.2 - 2.1
Circadian acrophase (clock time) - SR	p=0.35		p=0.07^	
Median [Q1, Q3]	02:16 [00:59, 03:55]	02:51 [01:45, 04:04]	02:03 [00:27, 02:28]	03:55 [02:33, 04:51]
Range	23:35 - 05:23	00:06 - 15:49	00:06 - 04:46	23:35 - 15:49
Physiologic chest surface temperature	p=1		p=1	
Yes	9 (100)	11 (91.7)	10 (100)	10 (90.9)
No	0 (0)	1 (8.3)	0 (0)	1 (9.1)
Circadian mesor (°C) - cosinor	p=0.58		p=0.0002***	
Median [Q1, Q3]	34.9 [34.5, 35.3]	34.6 [34.2, 35.4]	34.5 [34.3, 34.6]	35.4 [34.9, 35.6]
Range	34.1 - 36.0	33.8 - 36.4	34.0 - 35.2	33.8 - 36.4
Circadian amplitude (°C) - cosinor	p=0.05*		p=0.64	
Median [Q1, Q3]	0.9 [0.7, 1.0]	1.2 [1.0, 1.4]	1.2 [0.9, 1.4]	1.0 [0.8, 1.1]
Range	0.5 - 1.3	0.3 - 2.4	0.5 - 1.7	0.3 - 2.4
Circadian acrophase (clock time) - cosinor	p=0.17		p=0.75	
Median [Q1, Q3]	03:10 [02:25, 04:50]	02:58 [02:17, 03:44]	02:22 [02:13, 03:25]	02:58 [02:17, 03:44]
Range	01:40 - 06:20	22:30 - 04:05	22:30 - 06:20	22:30 - 04:05
**Synchronization signals**	**N=18**	**N=15**	**N=15**	**N=18**
Light acrophase (clock time)	p=0.56		p=0.70	
Median [Q1, Q3]	13:15 [12:23, 13:47]	13:30 [12:44, 14:39]	13:02 [12:45, 13:56]	13:33 [12:23, 14:34]
Range	11:32 - 15:02	10:46 - 15:26	10:46 - 15:02	11:02 - 15:26

cancers

Table S2. Comparison of rest-activity and chest surface temperature circadian parameters, synchronization signals, cortisol rhythm and DLMO between the (I<O)$_{high}$ patients and the controls older than 40 years. When not specified the data are presented as Number of subjects (%).

	Controls Age ≥ 40 years	Cancer patients (I<O)$_{high}$
**Rest-activity circadian rhythm**	**N=15**	**N=12**
I<O over study week (%)	p=0.15	
Median [Q1, Q3]	98.3 [97.5, 99.4]	97.3 [96.5, 98.6]
Range	95.0 - 100.0	93.3 - 99.0
Median of moderate-to-high activity (acc/min)	p<0.0001****	
Median [Q1, Q3]	185.3 [172.9, 199.6]	106.1 [86.6, 131.2]
Range	142.7 - 210.0	47.8 - 169.2
Circadian amplitude (acc/min)	p=0.37	
Median [Q1, Q3]	67.5 [63.0, 78.1]	63.9 [59.9, 71.7]
Range	39.5 - 101.5	46.8 - 91.1
Circadian acrophase (clock time)	p=0.35	
Median [Q1, Q3]	14:00 [12:29, 14:50]	13:13 [12:13, 14:25]
Range	11:43 - 16:01	10:20 - 16:36
P1-1	p=0.01*	
Median [Q1, Q3]	0.96 [0.94, 0.97]	0.94 [0.93, 0.94]
Range	0.93 - 0.98	0.91 - 0.97
r24	p=0.54	
Median [Q1, Q3]	0.39 [0.32, 0.45]	0.36 [0.33, 0.43]
Range	0.20 - 0.55	0.25 - 0.54
Rhythm Index	p=0.60	
Median [Q1, Q3]	0.74 [0.58, 0.80]	0.70 [0.63, 0.76]
Range	0.48 - 0.92	0.54 - 0.79
Rest duration (hours)	p=0.62	
Median [Q1, Q3]	8.3 [7.5, 9.4]	8.4 [7.5, 10.7]
Range	5.3 - 12.9	6.4 - 12.6
Center-of-rest time (clock time)	p=0.16	
Median [Q1, Q3]	02:20 [01:48, 03:08]	03:05 [02:17, 03:50]
Range	01:10 - 03:50	00:55 - 06:50
**Chest surface temperature circadian rhythm**	**N=12**	**N=11**
Circadian amplitude (°C) - SR	p=0.18	
Median [Q1, Q3]	1.1 [0.6, 1.3]	0.9 [0.6, 1.1]
Range	0.2 - 2.1	0.3 - 1.4
Circadian acrophase (clock time) - SR	p=0.20	
Median [Q1, Q3]	02:51 [01:45, 04:04]	01:33 [23:55, 03:09]
Range	00:06 - 15:49	20:17 - 08:44
Physiologic chest surface temperature	p=0.59	
Yes	11 (91.7)	9 (81.8)
No	1 (8.3)	2 (18.2)
Circadian mesor (°C) - cosinor	p=0.27	
Median [Q1, Q3]	34.6 [34.2, 35.4]	35.1 [34.9, 35.4]
Range	33.8 - 36.4	33.9 - 35.9
Circadian amplitude (°C) - cosinor	p=0.12	
Median [Q1, Q3]	1.2 [1.0, 1.4]	1.0 [0.7, 1.1]
Range	0.3 - 2.4	0.5 - 1.5
Circadian acrophase (clock time) - cosinor	p=0.40	
Median [Q1, Q3]	02:58 [02:17, 03:44]	03:10 [02:16, 04:03]
Range	22:30 - 04:05	18:50 - 04:40
**Synchronization signals**	**N=15**	**N=11**
Light acrophase (clock time)	p=0.11	
Median [Q1, Q3]	13:30 [12:44, 14:39]	12:24 [11:32, 13:17]
Range	10:46 - 15:26	10:23 - 14:46
Median wake-up time (clock time) - diary	p=0.06^	
Median [Q1, Q3]	06:10 [05:45, 07:00]	07:10 [06:27, 08:00]
Range	05:00 - 07:45	05:00 - 10:00
Median breakfast time (clock time) - diary	p=0.15	

Median [Q1, Q3]	08:00 [07:22, 08:34]	08:40 [08:02, 09:07]
Range	06:30 - 09:30	07:00 - 10:30
Median lunch time (clock time) - diary	p=0.13	
Median [Q1, Q3]	12:52 [12:34, 13:11]	13:00 [13:00, 13:15]
Range	09:45 - 13:45	12:35 - 13:37
Median dinner time (clock time) - diary	p=0.02*	
Median [Q1, Q3]	19:00 [18:30, 19:30]	18:15 [17:45, 18:35]
Range	17:15 - 20:15	17:00 - 19:30
Median retiring time (clock time) - diary	p=0.20	
Median [Q1, Q3]	23:00 [22:38, 23:42]	22:30 [22:17, 22:48]
Range	21:15 - 00:15	19:00 - 01:00
Median wake-up time (clock time) - HMM	p=0.008**	
Median [Q1, Q3]	06:31 [05:30, 07:16]	07:41 [07:13, 08:25]
Range	04:01 - 07:53	05:22 - 10:30
Median sleep onset (clock time) - HMM	p=0.10^	
Median [Q1, Q3]	22:52 [22:30, 23:28]	22:16 [20:41, 23:00]
Range	20:16 - 00:30	16:50 - 00:00
**Salivary cortisol circadian rhythm**	**N=13**	**N=7**
Circadian mesor (mol/L)	p=0.18	
Median [Q1, Q3]	4.4 [3.3, 5.2]	5.6 [4.4, 7.0]
Range	3.1 - 7.2	3.1 - 9.4
Circadian amplitude (mol/L)	p=0.01*	
Median [Q1, Q3]	3.3 [2.8, 3.9]	5.2 [3.9, 6.3]
Range	1.9 - 5.5	3.4 - 6.5
Circadian acrophase (clock time)	p=0.31	
Median [Q1, Q3]	08:47 [08:13, 09:09]	07:53 [07:11, 09:11]
Range	05:27 - 11:39	06:49 - 10:09
**Dim Light Melatonin Onset**	**N=10**	**N=7**
DLMO (clock time)	p=0.09^	
Median [Q1, Q3]	20:30 [19:21, 21:12]	21:44 [20:55, 22:16]
Range	17:22 - 21:33	18:23 - 23:32

Abbreviations: SR = Spectrum Resampling; HMM = Hidden Markov Model; DLMO = Dim Light Melatonin Onset. Comparisons between controls aged 40 years or more and cancer patients with $(I<O)_{high}$ were done with two-tailed Welch's t-tests for continuous variables and Fisher's exact test for categorical variables. The corresponding p-values are shown for each parameter as follows: ****p<0.0001; ** 0.001< p ≤0.01; * 0.01< p ≤0.05; ^ 0.05< p ≤0.1.
